# Three-dimensional positional relationship between impacted mandibular third molars and the mandibular canal

**DOI:** 10.1186/s12903-023-03548-0

**Published:** 2023-11-04

**Authors:** Yun Yang, Dong-Yu Bao, Can Ni, Zhen Li

**Affiliations:** 1 Department of Stomatology, Nanjing Drum Tower Hospital, the Affiliated Hospital of Nanjing University Medical School, Nanjing, 210008 Jiangsu China; 2 Department of Periodontics, Nanjing Stomatological Hospital, Medical School of Nanjing University, Nanjing, 210008 Jiangsu China; 3 Department of Implantology, Nanjing Stomatological Hospital, Medical School of Nanjing University, Nanjing, 210008 Jiangsu China No. 30 of Central Road, Xuanwu District,

**Keywords:** Mandibular canal, Impacted mandibular third molar, Cone beam CT, Surface tomogram, Nerve injury

## Abstract

**Objective:**

To observe the three-dimensional positional relationship between impacted mandibular third molars (IMTMs) and mandibular canal close contacts using cone beam computed tomography (CBCT).

**Methods:**

A total of 101 patients with IMTMs were selected who met the diagnostic criteria for 142 teeth (no bone wall imaging area between IMTMs and the mandibular canal, a high-density bone cortical imaging area only, or a ≦1 mm bone imaging area). The parameters of the rotating CBCT anode were set as follows: 110 kV, 40–50 mA; the focal point and exposure field were set as 0.3 mmh and a high-resolution zoom, respectively; the exposure time and image layer thickness were set as 5.4 s and 0.25 mm. Three-dimensional reconstruction was performed, and the position of the mandibular canal through the IMTM area was observed continuously from the coronal, horizontal and sagittal planes.

**Results:**

We found that the mandibular canal was interrupted below the third molar (TM) in 85 cases, accounting for 59.86% of all cases. The mandibular canal was located below the buccal and lingual curvatures in 33 and 19 cases, respectively, accounting for 23.23% and 19%. In addition, a small number of mandibular canals were also located on the buccal side of the mandibular molars (2.82%). We also found one case of direct insertion of the mandibular third molar (MTM) into the mandibular canal. In addition, the mandibular canal passed through the IMTM region with 125 close contacts at the roots (88.03%); 14 mandibular canals were in contact with all teeth and 3 were in contact with the crown.

**Conclusion:**

The use of CBCT can provide a dynamic and comprehensive understanding of the three-dimensional positional relationship of the mandibular alveolar nerve canal passing through the IMTM area, providing a high clinical reference value when extracting IMTMs and reducing the risk of injury to the inferior alveolar nerve.

## Introduction

Impacted mandibular third molars (IMTMs) refer to those third molars (TMs) in the lower jaw that are in close contact with or exert pressure on adjacent anatomical structures, such as the mandibular canal or the second molar. The prevalence of IMTMs varies between different populations, ranging from 9.5% to 68% [1]. Extraction of IMTMs is the most common procedure performed in oral clinics [2]. The extraction of mandibular impacted wisdom teeth is a difficult procedure, particularly for complex low-impacted wisdom teeth, and the extraction of IM wisdom teeth often results in complications, such as loosening or damage to adjacent teeth, dry sockets and broken roots [3]. Another critical concern during the extraction of IMTMs is the potential risk of damaging the inferior alveolar nerve (IAN) due to its close proximity to the IMTMs. The incidence of nerve injury is reported to be 0.5%–8%, so it is extremely important to understand the position of IMTMs near the mandibular canal [4]. Surface tomography is often used to understand the position of IMTMs, relative to the mandibular canal, before removal of the former in clinical practice. However, the two-dimensional images generated by surface tomography may be inaccurate for determining the close contact between the two. Additional X-rays can only image the tooth in two dimensions, not three. This leads to an inaccurate assessment of the position of the roots and mandibular canal, which, in turn, will seriously affect the surgeon's surgical judgment and cause more complications and pain for the patient [5].

With the innovation of imaging technology, cone beam computed tomography (CBCT) was developed. This technique relies on CB volumetric imaging technology to observe lesion sites inside the oral cavity and diagnose the disease using CT. Its positioning accuracy is higher and images are clearer than conventional X-ray diagnostic methods [6]. In addition, the scanned lesion site can be presented as a three-dimensional image, which has a lower risk of overlapping oral structures and a higher disease detection rate than conventional two-dimensional images [7].

Applications in implantology, endodontics, orthodontics and oral and maxillofacial surgery have been reported [8–11]. CBCT is specifically recommended as the method of choice when a three-dimensional view of the affected mandibular TM and adjacent anatomical structures, such as the IAN canal and lingual cortex, is required [4, 8, 12–15].

The existing literature has extensively studied the relationship between impacted mandibular third molars (ITMs) and the mandibular canal, highlighting the importance of accurate preoperative assessments to prevent nerve injuries. However, there remains a need for further investigation specifically focusing on IMTMs to provide a comprehensive understanding of their distinct characteristics and spatial positioning in relation to the mandibular canal [16–18]. To expand the sample size of this research topic, the current paper observed the imaging data of 101 cases of IMTMs in close contact with the mandibular canal by CBCT and dynamically analysed the location of the IAN across the IMTM region to provide an additional clinical reference for the extraction of IMTMs.

## Methods and materials

### Clinical information

Patients who visited the Department of Stomatology of Nanjing Tongren Hospital from February 2020 to February 2021 and requested extraction of IMTMs were selected, as well as those who were found to have contact or overlap between IMTMs and the mandibular canal after performing curved tomograms, followed by CBCT examinations. A total of 101 patients with 142 teeth were selected for whom the mandibular obstructed TM was in close contact with the mandibular canal. In these cases, IMTMs and the mandibular canal were in close contact mainly as follows: there was no bone wall imaging area between IMTMs and the mandibular canal, only a high-density bone cortical imaging area, or a ≦1 mm bone imaging area. The three-dimensional positional relationship between IMTMs and the mandibular canal was dynamically observed in 53 cases (74 teeth) in men and 48 cases (68 teeth) in women. The exclusion criteria for patients were as follows: after taking curved tomographs and finding that the IMTM was not in close contact with the mandibular neural nerve, the patient was excluded.

The patients' ages ranged from 16–72 years old, with an average of 35.42 years.

### Imaging equipment and method

To conduct CBCT, the NewTom VGI Tomograph (NewTom, Verona, Italy), which uses the Frankfurt plane as the reference plane and can be rotated 360°, was used. The X-ray CB automatically adjusts the radiation dose according to the patient’s volume. The parameters of the rotating anode were as follows: 110 kV, 40–50 mA; 0.3 mmh and a high-resolution zoom were used for focus and exposure field, respectively; 5.4 s and 0.25 mm were used for exposure time and image layer thickness, respectively.

**Fig. 1:**
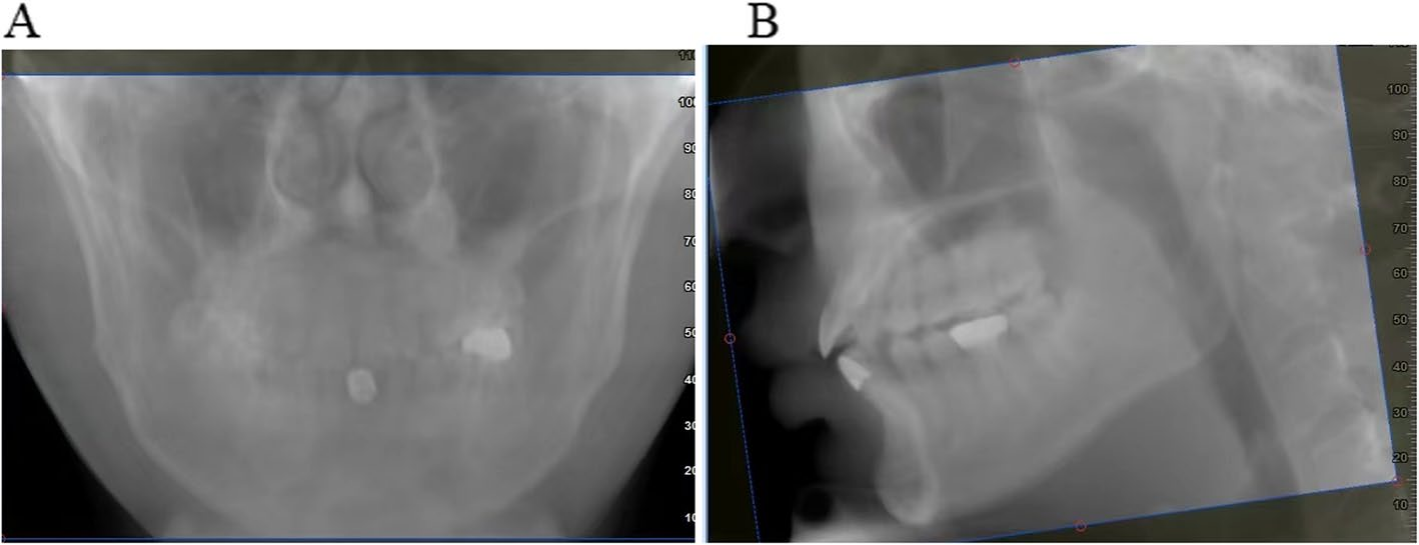
During the reconstruction of study data, fine adjustments are made to the reference plane (**A**) and the mid-sagittal plane (**B**)

For precise positioning of the patient, we instructed them to maintain an upright position with their eyes looking straight ahead. The Frankfurt horizontal plane, which runs from the external auditory meatus to the infraorbital rim, was aligned parallel to the ground. We utilized a head fixation device to secure the patient's head in this position. During image capture, we ensured that the long axis of the patient's head was perpendicular to the ground. After the initial capture, during the reconstruction of the study data, we performed fine adjustments to the reference plane to ensure that the Frankfurt horizontal plane remained parallel to the ground and that the midsagittal plane aligned with the vertical axis. (See Fig. 1 for an illustration of these adjustments during the reconstruction of study data).

### *Analysismethod*

The images were reconstructed in three dimensions using the NNT software (v.*, QR SRL, Verona, Italy), and the position of the mandibular canal passing through the IMTM area was observed continuously from the coronal, horizontal and sagittal planes, respectively. In the adjustment of the reference lines in the reconstructed panoramic view to adjust the cross-sectional image, vertical reference lines was used (by adjusting the reference plane to ensure that the Frankfurt horizontal plane was parallel to the horizontal plane, the mid-sagittal plane was parallel to the sagittal plane, and the cross-sectional views were cut vertically across the horizontal plane).

## Results

### Position relationship between the mandibular canal and the mandibular third interceptive molar

**Fig. 2:**
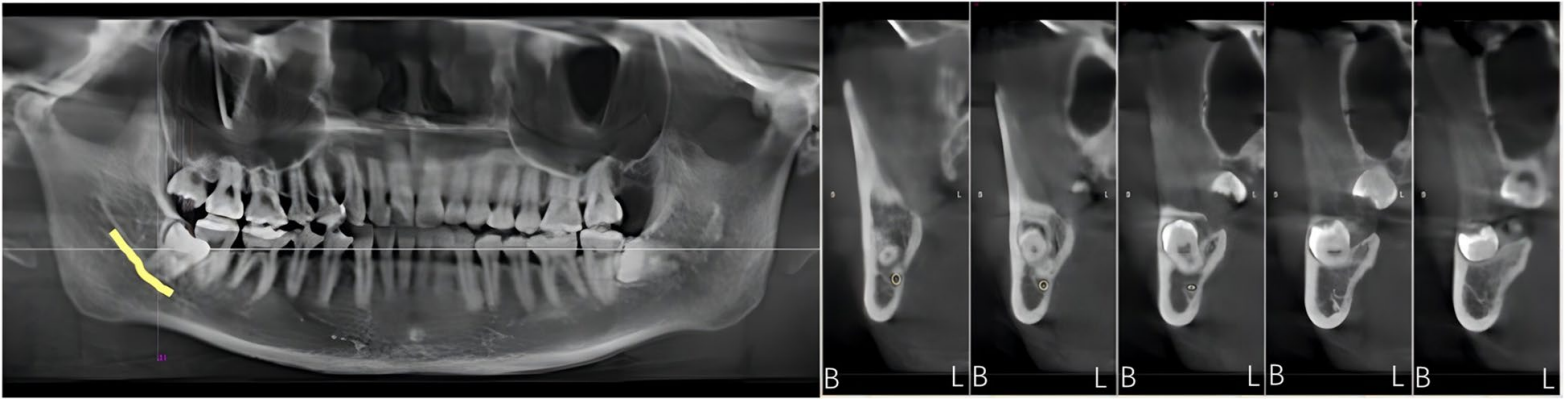
The mandibular canal (yellow color) passes under the tooth root. "B" for buccal side, "L" for lingual side

**Fig. 3:**
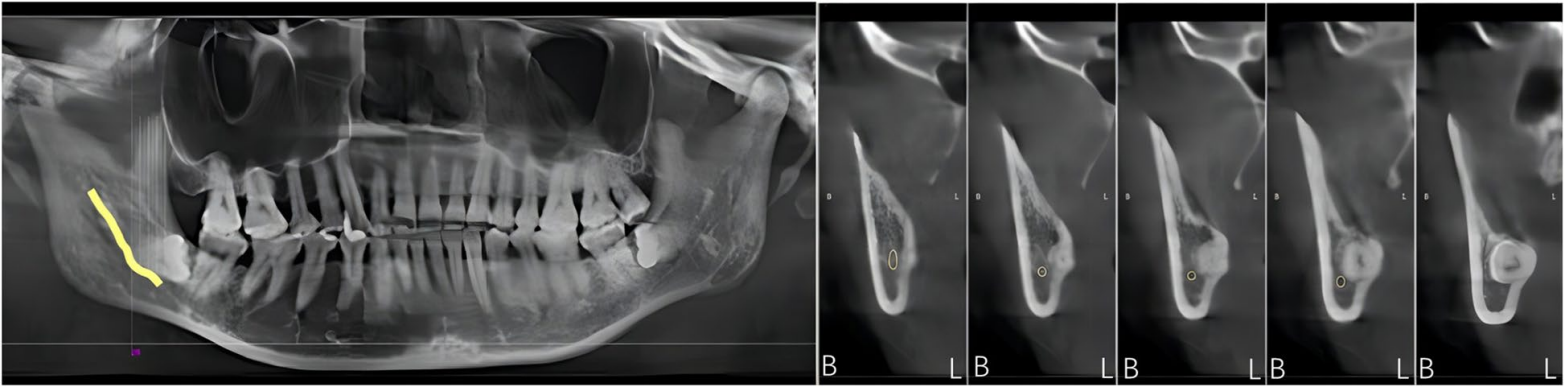
The mandibular canal (yellow color) enters from the buccal side of the apical region and leaves under the crown. "B" for buccal side, "L" for lingual side

**Fig. 4:**
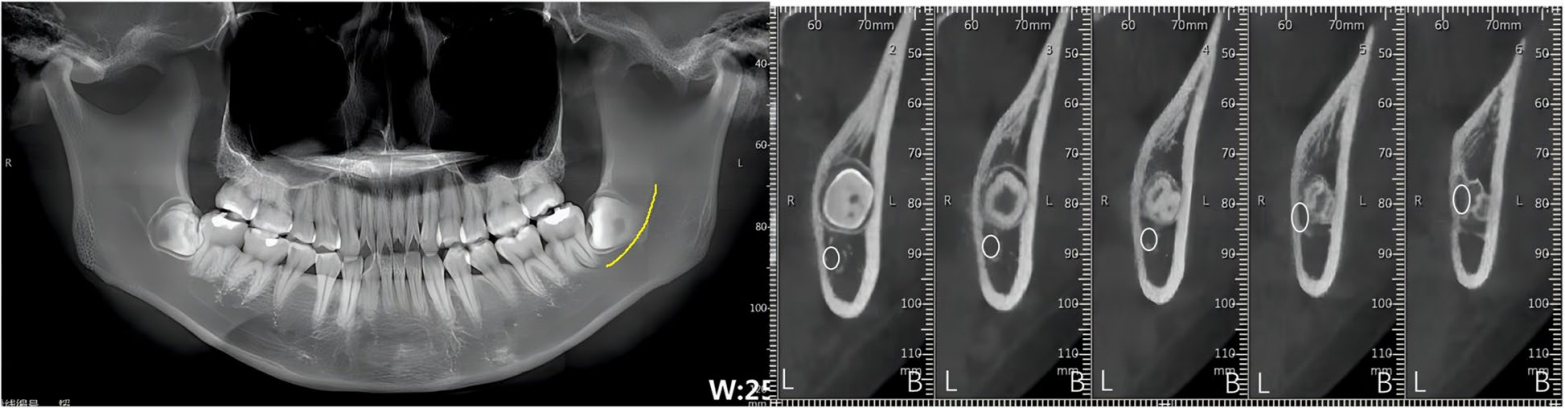
The mandibular canal (yellow color) enters from the lingual side of the apical region and leaves under the crown. "B" for buccal side, "L" for lingual side. The white circle represents the mandibular canal

**Fig. 5:**
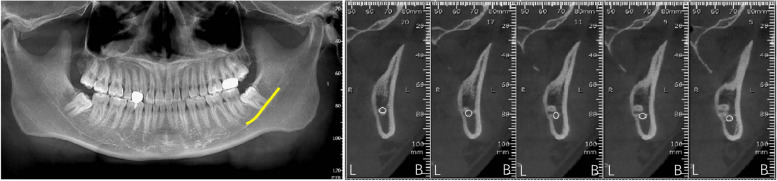
The mandibular canal (yellow color) passes buccally from the apical region.. "B" for buccal side, "L" for lingual side

**Fig. 6:**
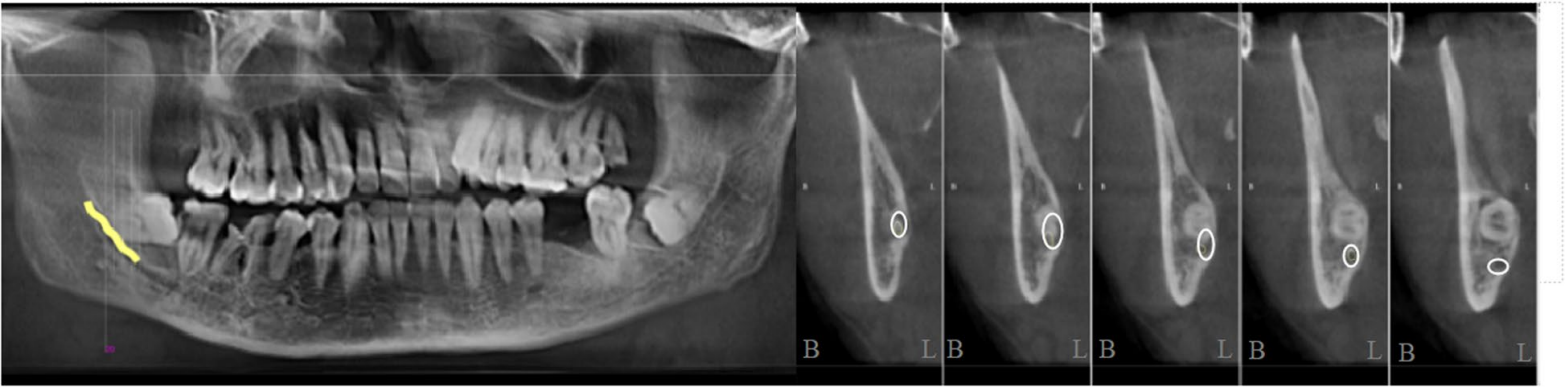
The root of the tooth enters the mandibular canal (yellow color)

By analysing the positional relationship between the mandibular canal and the mandibular third interrupted molar (from the position of the mandibular canal into the IMTM region to the exit position) to determine the risk of nerve injury during extraction of the interrupted TM, it was found that the mandibular canal passed through below the interrupted TM in 85 cases, accounting for 59.86% of all the study cases (Fig. 2). The mandibular canal was located below the buccal lateral turn of the interrupted molar in 33 cases, accounting for 23.23% (Fig. 3) and below the lingual lateral turn of the IMTM in 19 cases, accounting for 13.38% (Fig. 4). In addition, the mandibular canal was located on the buccal side of the interrupted molar in a small number of cases (2.82%, Fig. 5). We also found one patient with an IMTM seated into the mandibular canal (Fig. 6) (Table 1).

**Table 1:** Positional relationship between mandibular canal and 142 IMTM

	Inferior	Buccal	Buccal towards inferior	lingual towards inferior	Intracanal
Position relative to IMTM	85(59.86%)	4(2.82%)	33(23.23%)	19(13.38%)	1(0.70%)

### Analysis of the close contact sites between the mandibular canal and the IMTMs

**Fig. 7:**
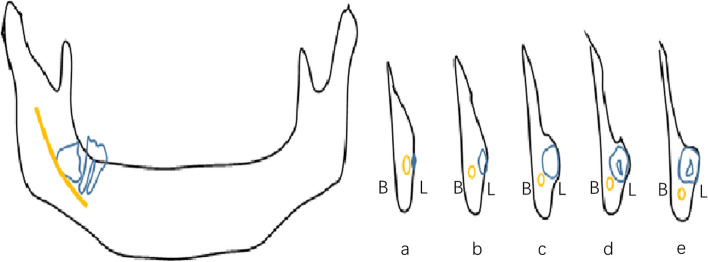
The close contact sites between the mandibular canal and the mandibular blocked third molars. "B" for buccal side, "L" for lingual side. Schematically, one can observe the position of the mandibular neural tube (yellow) in relation to the mandibular third molar (blue) as it travels from posterior to anterior (a → e)

Where the mandibular canal passed through the IMTM area, there were 125 close contact sites at the root, accounting for 88.03%; 14 mandibular canals contacted all of the teeth and 3 contacted the crown (Table 2). The close contact sites between the mandibular canal and the mandibular-blocked TMs are shown in Fig. 7.

**Table 2:** Positional relationship between mandibular canal and 142 IMTM

	Root	All teeth	Crown
Position relative to IMTM	125(88.03%)	14(9.86%)	3(2.11%)

## Discussion

IAN injury is one of the serious complications of IMTM extraction, and the closer the mandibular canal is to the TM, the greater the chance of injury. Accurate knowledge of the position of the IMTM near the mandibular canal before extraction is essential and will determine the extraction procedure [19]. Several methods have been proposed to reduce or eliminate this obstacle, such as orthodontically assisted extraction [20, 21], extraction of the second molar, or crown amputation [22, 23]. Liu Yaguo [24] observed and measured the positional relationship and distance between the mandibular canal and the proximal mesial and distal mesial root apices on a coronal sawing section of the middle portion of the adult mandibular third molar crown and categorised the positional relationship into three categories: (1) medial to the root apices, accounting for 5.2%; (2) lateral to the root apices, accounting for 8.3%; (3) inferior to the root apices, accounting for 86.5%. For distance measurements, the findings were as follows: (1) located at a medial distance of 0.4 ± 0.2 (0.3 ~ 0.8) mm; (2) the distance located on the lateral side of the root tip was 0.4 ± 0.2 (0.2 ~ 0.5) mm; (3) the distance located below the root tip was 4.9 ± 3.0 (1.0 ~ 11.5) mm. In the present study, the study of the relationship between the mandibular canal and the TM with solid specimens was affected by the number of specimens, and it was not possible to obtain a large volume of case data concerning close contact between the mandibular canal and the TM. However, the extent of the positional relationship, as well as the distance between the mandibular canal and the proximal and distal mesial root sections of the TM, could be observed.

Curved surface tomography is the main method for preoperative clinical assessment of the position and distance of the mandibular canal from the TM because of its rapidity, simplicity, low cost and low radiation exposure, and because it can show all the teeth and jaws at the same time. However, because it presents the three-dimensional anatomical relationship in a two-dimensional plane [25], it cannot accurately show the buccolingual–lingual positioning relationship between the nerve canal and the root of the tooth and may produce image magnification and distortion, based on the imaging technique used. Liang RQ et al. [26, 27] classified the relationship between the IMTM and mandibular canal using panoramic radiographs into four categories: Class A – leaving, where the tooth root is separated from the mandibular canal and located ≥ 1 mm from the upper edge of the mandibular canal; Class B – contact, where the tooth root is < 1 mm from the upper edge of the mandibular canal; Class C – overlap, where the tooth root is located at the upper edge of the mandibular canal without reaching the lower edge; Class D – cross, where the tooth root is located below the lower edge of the mandibular canal. The risk of injury to the IAN during tooth extraction was considered high in categories C and D. The accuracy of the panoramic film was poor, however, due to the defects of projection-angle deviation, artefacts, image overlap and distortion; the rate of clear visualisation of the mandibular canal in the TM segment was 60.73%, the rate of a blurred upper edge was 26.44% and the rate of blurred upper and lower edges was 12.84% [28]. Peng Min et al [29] concluded that the sensitivity of panoramic film to evaluate the risk of IAN injury during extraction of IMTMs was 57%. Therefore, when the mandibular canal is in close contact with an IMTM, it is inaccurate to refer only to curved tomographic film to understand the positional relationship between the mandibular canal and the IMTM in three dimensions, and injury to the IAN during tooth extraction will be inevitable [30].

Currently, CBCT is used clinically in cases of contact and overlap between IMTMs and the mandibular canal via curved tomogram to evaluate the positional relationship between the mandibular canal and IMTMs. The relationship between the mandibular canal and IMTM root position is classified into four categories: lingual, buccal, inter-root and subrogated and used to assess the risk of IAN injury [31–33]. The digital measurement software that comes with CBCT is used to measure the root and mandibular length, width and height of the nerve canal contact to provide an accurate, quantitative guide for surgical extraction procedures [34]. Unlike curved tomograms, CBCT images do not influence the surgical extraction protocol; however, they provide clear visualization of the relationship between the mandibular canal and an impacted third molar (ITM) [35]. In this way, the risk of IAN injury during the extraction of IMTMs can be better predicted [36]. The mandibular canal-IMTM contact and the increased area of the mandibular canal are factors that contribute to an elevated risk of IAN injury [18]. Rood and Sheehab distinguished between four radiological signs observed in the root (darkening, tilting and narrowing of the root and apical bifurcation) and three additional signs observed in the root canal (shunting, narrowing and interruption of the white line of the root canal) [37]. However, the absence of these signs does not guarantee the absence of close contact. This means that when the roots project excessively into the mandibular canal in panoramic images, particularly when one or more signs are present, additional radiographic examination may be needed to elucidate the three-dimensional relationship between these two structures.

In our study, CBCT analysis revealed that the IAN was further below and buccal to the mandibular obstructed TM, while the relationship between the TM and the mandibular canal was not only point-to-point, nor was it only the root being in close contact with the mandibular canal; a few TMs were in close contact with the nerve canal by the whole tooth or the crown, which was due to the different tooth obstruction positions, i.e. vertical obstruction, different angles of inclined obstruction, horizontal obstruction and even inverted obstruction. Three imaging distance manifestations of the degree of close contact between the mandibular canal and IMTM were observed: absence of a bone wall image, presence of only a high-density cortical bone image, and existence of both bone density and a small amount of bone cancellous image. Clinically, the site and degree of close contact between the mandibular canal and IMTMs must be dynamically observed to obtain complete imaging information of the mandibular canal passing through the IMTM area and to determine a perfect surgical plan for tooth extraction. During tooth extraction, whether operating on the crown, root, lingual or buccal side, it is clear that the mandibular canal is there to avoid blind operation to avoid and reduce the risk of nerve injury.

## Conclusion

In conclusion, this study provides a novel and clinically significant investigation of the three-dimensional positional relationship between impacted mandibular third molars and the mandibular canal using CBCT. The use of CBCT offers a more accurate assessment, enhancing the understanding of IMTM extraction and reducing the risk of nerve injury during procedures. This research contributes valuable insights for dental practitioners, improving surgical planning and patient outcomes.

## Data Availability

All data generated or analysed during this study are included in this article. Further enquiries can be directed to the corresponding author.

## References

[1] Abu-Hussein Muhamad, Watted Nezar, Abdulgani Azzaldeen. Prevalence of Impacted Mandibular Third Molars in Population of Arab Israeli: A Retrospective Study IOSR J Dent Med Sci 2016,(15): 80-9.

[2] Jaroń A, Preuss O, Konkol B, Trybek G (2021). Quality of Life of Patients after Kinesio Tape Applications Following Impacted Mandibular Third Molar Surgeries. J Clin Med.

[3] Lin-Lin J, Yan Hu (2022). Clinical use of oral and maxillofacial cone beam CT in the extraction of complex mandibular low obstructed wisdom teeth. Chin Med Device Inform.

[4] Korkmaz YT, Kayıpmaz S, Senel FC, Atasoy KT, Gumrukcu Z (2017). Does additional cone beam computed tomography decrease the risk of inferior alveolar nerve injury in high-risk cases undergoing third molar surgery?Does CBCT decrease the risk of IAN injury?. Int J Oral Maxillofac Surg.

[5] Perschbacher S (2012). Interpretation of panoramic radiographs[J]. Aust Dent J.

[6] Latt M, Chewpreecha P, Wongsirichat N (2015). Prediction of difficulty in impacted lower third molars extraction: Review of literature. M Dent J.

[7] Ghaeminia H, Meijer GJ, Soehardi A (2009). Position of the impacted third molar in relation to the mandibular canal. Diagnostic accuracy of cone beam computed tomography compared with panoramic radiography. Int J Oral Maxillofacial Surg.

[8] Vinay Kumar K, Maloth K, Ashwini P (2017). Objectivity and reliability of panoramic radiographic signs and cone-beam computed tomography in the assessment of a superimposed relationship between the impacted mandibular third molars and mandibular nerve: A comparative study. Indian Acad Oral Maxillofac Radiol.

[9] Suomalainen A, Ventä I, Mattila M (2010). Reliability of CBCT and other radiographic methods in preoperative evaluation of lower third molars. Oral Surg Oral Med Oral Pathol Oral Radiol Endod.

[10] Tantanapornkul W, Okouchi K, Fujiwara Y (2007). Acomparative study of cone-beam computed tomography and conventional panoramic radiography in assessing the topographic relationship between the mandibular canal and impacted third molars. Oral Surg Oral Med Oral Pathol Oral Radiol Endod.

[11] Manoj Kumar S, Chandra Mouli PE, Kailasam S (2011). Applications of cone beam computed tomography in dentistry. Indian Acad Oral Maxillofac Radiol.

[12] Atieh MA (2010). Diagnostic accuracy of panoramic radiography in determining relationship between inferior alveolar nerve and mandibular third molar. J Oral Maxillofac Surg.

[13] Flygare L, Ohman A (2008). Preoperative imaging procedures for lower wisdom teeth removal. Clin Oral Investig.

[14] Baranwal A, Srivastava A, Chaurasia A (2015). Cone beam computed tomography: A new dimension of imaging with basics and clinical applications in dentistry. Int J Maxillofac Imaging.

[15] Patel PS, Shah JS, Dudhia BB  (2020). Comparison of panoramic radiograph and cone beam computed tomography findings for impacted mandibular third molar root and inferior alveolar nerve canal relation. Indian J Dent Res.

[16] Alkhader M, Jarab F (2016). Visibility of the mandibular canal on cross-sectional CBCT images at impacted mandibular third molar sites. Biotechnol Equip.

[17] Hongping C (2022). Discussion on the application of cone beam CT in the clinical diagnosis and treatment of stomatology. Modern Med Imaging.

[18] Peker I, Sarikir C, Alkurt MT (2014). Panoramic radiography and cone-beam computed tomography findings in preoperative examination of impacted mandibular third molars. BMC Oral Health.

[19] Xue-Tao W, Tsung Li, Xiang-Chun Li (2020). The effect of CBCT on the choice of surgical procedure for mandibular third molar. Dental Res.

[20] Ma ZG, Xie QY, Yang C (2013). An orthodontic technique for minimally invasive extraction of impacted lower third molar. J Oral Maxillofac Surg.

[21] Zhou J, Hong H, Zhou H (2021). Orthodontic extraction of a high-risk impacted mandibular third molar contacting the inferior alveolar nerve with the aid of a ramus mini-screw. Quintessence Int.

[22] Wu XC, Li Y, Zhao JJ (2019). Clinical evaluation for coronectomy of the impacted mandibular third molars in close proximity to inferior alveolar nerve. Shanghai Kou Qiang Yi Xue.

[23] Monaco G, D'Ambrosio M, De Santis G (2019). Coronectomy: A Surgical Option for Impacted Third Molars in Close Proximity to the Inferior Alveolar Nerve-A 5-Year Follow-Up Study. J Oral Maxillofac Surg.

[24] Liu YAGUO. Position of the mandibular third molar root tip in relation to the mandibular canal. J Clin Anat, 1986, 4(4): 242–243.

[25] Qin LY, Zhou ZW (2019). Progress of research on the accuracy of the determination of mandibular wisdom teeth and inferior alveolar nerve adjacent relationship by panoramic film. Oral Med Res.

[26] Liang RQ, Pan WC (2016). Panoramic film image analysis of the risk of injury to the inferior alveolar nerve by extraction of mandibular interrupted third molar. J Right River Sch Ethnic Med.

[27] Liang RQ, Yu F, Wang DCC (2016). Clinical analysis of the relationship between the visualization of mandibular canal in panoramic film and mandibular obstructed third molar. Gansu Med.

[28] van der Stelt PF (2016). Panoramische röntgenopnamen in de tandheelkundige diagnostiek [Panoramic radiographs in dental diagnostics]. Ned Tijdschr Tandheelkd.

[29] Min P, Kun T, Zhimin Z (2009). Application of panoramic film in predicting inferior alveolar nerve injury during extraction of mandibular third obstructive molar. J Pract Radiol.

[30] Sarikov R, Juodzbalys G (2014). Inferior alveolar nerve injury after mandibular third molar extraction: a literature review. J Oral Maxillofac Res.

[31] Ghaeminia H, Meijer GJ, Soehardi A (2011). The use of cone beam CT for the removal of wisdom teeth changes the surgical approach compared with panormic radiography: a pilot study[J]. J Oral Maxillofac Surg.

[32] Guangzhou Xu, Chi Y, Xindong F (2014). Classification and clinical significance of mandibular obstructive third molars with reference to the mandibular nerve canal. Chin J Oral Maxillofacial Surg.

[33] Lanzhu H, Tie-Mei W, Zheng H (2014). Application of CBCT in preoperative risk assessment of mandibular obstructed third molar extraction[J]. J Clin Dent.

[34] Yusheng Z, Chunyan Z, Qiang Z (2016). Application of CBCT in the study of the anatomical relationship between the inferior alveolar nerve canal and mandibular obstructive third molars [J]. Pract Clin Med.

[35] Araujo GTT, Peralta-Mamani M, Silva AFMD (2019). Influence of cone beam computed tomography versus panoramic radiography on the surgical technique of third molar removal: a systematic review. Int J Oral Maxillofac Surg.

[36] Reia VCB, de Toledo T-A, Peralta-Mamani M (2021). Diagnostic accuracy of CBCT compared to panoramic radiography in predicting IAN exposure: a systematic review and meta-analysis. Clin Oral Investig.

[37] Rood JP, Shehab BA (1990). The radiological prediction of inferior alveolar nerve injury during third molar surgery. Br J Oral Maxillofac Surg.

